# Histological Characterization of the Dicer1 Mutant Zebrafish Retina

**DOI:** 10.1155/2015/309510

**Published:** 2015-06-01

**Authors:** Saeed Akhtar, Sarita Rani Patnaik, Rakesh Kotapati Raghupathy, Turki M. Al-Mubrad, John A. Craft, Xinhua Shu

**Affiliations:** ^1^Cornea Research Chair, Department of Optometry, King Saud University, P.O. Box 10219, Riyadh 11433, Saudi Arabia; ^2^Department of Life Sciences, Glasgow Caledonian University, Glasgow G4 0BA, UK

## Abstract

DICER1, a multidomain RNase III endoribonuclease, plays a critical role in microRNA (miRNA) and RNA-interference (RNAi) functional pathways. Loss of *Dicer1* affects different developmental processes. Dicer1 is essential for retinal development and maintenance. DICER1 was recently shown to have another function of silencing the toxicity of *Alu* RNAs in retinal pigment epithelium (RPE) cells, which are involved in the pathogenesis of age related macular degeneration. In this study, we characterized a *Dicer1* mutant fish line, which carries a nonsense mutation (W1457Ter) induced by N-ethyl-N-nitrosourea mutagenesis. Zebrafish DICER1 protein is highly conserved in the evolution. Zebrafish Dicer1 is expressed at the earliest stages of zebrafish development and persists into late developmental stages; it is widely expressed in adult tissues. Homozygous *Dicer1* mutant fish (DICER1^W1457Ter/W1457Ter^) have an arrest in early growth with significantly smaller eyes and are dead at 14–18 dpf. Heterozygous *Dicer1* mutant fish have similar retinal structure to that of control fish; the retinal pigment epithelium (RPE) cells are normal with no sign of degeneration at the age of 20 months.

## 1. Introduction

DICER1, the RNase III enzyme, plays a central role in processing the long double stranded RNA (dsRNA) into small RNA molecules, including microRNAs (miRNAs) and small interfering RNAs (siRNAs) [1]. Both miRNAs and siRNAs regulate gene expression through assembling an RNA-induced silencing complex and consequently reducing the levels of functional protein within cells [1, 2]. DICER1 contains different functional domains including N-terminal helicase, DUF283, PAZ, two RNase III, and one dsRNA-binding domain [2]. The helicase domain is necessary for siRNA processing, producing endogenous siRNAs, the PAZ and RNase III domains function in RNA binding and cleavage to produce 2-nt 3′ overhangs and the dsRNA-binding domain has a role for dsRNA and cleavage [3, 4]. The role of DUF283 is unknown. The physiological role of DICER1 is indicated by several studies. Mutation in the* Dicer* gene of zebrafish or of* Caenorhabditis elegans* resulted in developmental arrest [5, 6]. Depletion of* Dicer1* in mouse caused early embryonic lethality [7], while tissue-specific conditional knock-outs of dicer 1 suggested the essential role of Dicer1 in development of various organs [8].

DICER1 plays an important role in regulating retinal development. Damiani et al. conditionally knocked out (CKO)* Dicer1* in mouse retinal progenitors using the Chx10Cre mouse and found abnormal retinal phenotypes after the second postnatal week [9]. Both homozygous and heterozygous* Dicer1* CKO mice exhibited decreased electroretinaogram (ERG) responses, with the lowest ERG response in the homozygous mouse eyes. The homozygous mice presented photoreceptor rosettes at age P16 which increased further by P45, along with displaced photoreceptor clusters. When mice reached the age of 3 months, the rosettes became small and disappeared, following the degeneration of photoreceptors. Georgi and Reh [10] conditionally knocked out Dicer1 using the *α*Pax6cre transgenic line and found that DICER1 depleted retinal progenitor cells did not progress to the late progenitor state, resulting in the lack of horizontal cells and amacrine cells and reduced photoreceptors at P5.* Dicer1* CKO mouse retinas are thinner compared to those of heterozygous littermates, because most of* Dicer1*-depleted cells die through apoptosis. Depletion of* Dicer1* in mature postmitotic rods resulted in fast degeneration with 90% of the outer nuclear layer disappearing at 14 weeks old [11]. The above data suggest* Dicer1* is required for the differentiation and survival of retinal cells.

Recently studies suggest DICER1 plays an important role in the pathogenesis of dry age-related macular degeneration (AMD). Dry AMD patients have a lower DICER1 level in retinal pigment epithelium (RPE) cells compared to that of healthy individuals. Deficiency of DICER1 in mouse RPE cells results in RPE degeneration induced by* Alu* RNA toxicity [12]. Further study discovered that* Alu* RNA accumulation could activate the NLRP3 inflammasome and trigger MyD88-mediated signaling, which lead to RPE cell degeneration [13]. Zebrafish has been widely used as a model to study retinal development and to understand the molecular mechanisms of retinal degeneration [14, 15]. To use zebrafish as a model to study the functional role of DICER1 in zebrafish retina, we examined the expression of* Dicer1* during development and in adult tissues. We also morphologically characterized the retina of aged* Dicer1* mutant zebrafish.

## 2. Materials and Methods

### 2.1. Ethics Statement

All the experiments using zebrafish were approved by the Animal Ethics and Welfare Committee, Department of Life Sciences, Glasgow Caledonian University. The project was approved by Home Office under a Project License PPL 60/4169.

### 2.2. Zebrafish Maintenance

AB strain zebrafish were obtained from the MRC Human Genetics Unit, Edinburgh, and maintained as an inbred stock in Glasgow Caledonian University Zebrafish Facility. Larvae and adult fish were kept in the ZEBTEC zebrafish housing system (Tecniplast) on a 14 : 10 h light/dark photoperiod at 28°C and were fed with brine shrimp twice a day.

### 2.3. Zebrafish* Dicer1* Mutant Line

The* Dicer1* mutant zebrafish line (Hu0894) was obtained from Wellcome Trust Sanger Institute and bred in our zebrafish facility. The Hu0894 mutant strain was induced by ENU (N-ethyl-N-nitrosourea) mutagenesis [16] and carried a premature stop mutation: G4371A (W1457Ter) referencing zebrafish* Dicer1* in the Ensembl website (ENSDART00000045881.5). Wienholds et al. [5] reported an ENU-induced mutant zebrafish strain (hu894) carrying a G4338A mutation (W1446X) and we compared the originally reported sequence (GenBank accession number AY386319) with the current referencing zebrafish* Dicer1* gene (GenBank accession number NM_001161453.2) and found that both mutant strains carried the same mutation.

### 2.4. Genotyping

Zebrafish tail clips were placed in 96-well plate. 25 *μ*L of 100% ethanol was added to each well and incubated at 80°C. 25 *μ*L of TE Tween-20 (with 5 mg/mL of Proteinase K) was added to each well and then incubated overnight at 56°C. The samples were then heated to 95°C for 15 min to inactivate Proteinase K. 75 *μ*L of dH_2_O was added to each sample.

Polymerase chain reaction (PCR) was performed using* Taq* DNA polymerase (NEB, M0273X) using the manufacturer's instructions. PCR reactions were set up in a 96-well PCR plate using the following protocol: 95°C for 2 min and then 35 cycles of 94°C for 30 sec, 58°C for 30 sec and 72°C for 60 sec, cooling to 4°C, and storage at 4°C. Aliquots of PCR were loaded onto 1% agarose gel and visualized with ethidium bromide staining. The primers specific for Dicer1 were 5′ TGCCATGTATGTGGCCATCCA 3′ and reverse: 5′ AACACAGTGCTGTCTGGAGGT 3′. Products of PCR reactions were sent for sequence determination.

### 2.5. Bioinformatic Analysis

Peptide sequences of Human (*Homo sapiens*: NP_803187.1), Cow (*Bos taurus*: NP_976235.1), Mouse (*Mus musculus*: NP_683750.2), Chicken (*Gallus gallus*: NP_001035555.1), Platanna (*Xenopus laevis*: NP_001163918.1), Zebrafish (*Danio rerio*: NP_001154925.1), Fruit fly (*Drosophila melanogaster*: NP_524453.1), Nematode (*Caenorhabditis elegans*: NP_498761.2), and Yeast (*Schizosaccharomyces pombe*: NP_588215.2) were obtained from NCBI and aligned using CLUSTALW program (http://www.ebi.ac.uk/Tools/msa/clustalw2/) and conserved regions were boxshaded using BoxShade 3.21 (http://www.ch.embnet.org/software/BOX_form.html). The primers were designed using NCBI primer design tool (http://www.ncbi.nlm.nih.gov/tools/primer-blast/) based on the coding sequences of Zebrafish* Dicer1* (NM_001161453.2) and* Beta-actin* (NM_131031.1).

### 2.6. Expression Analysis

Total RNA was extracted from different adult tissues and development stages of Zebrafish using Absolutely RNA Miniprep kit (Agilent) and reverse transcribed using Trancriptor high fidelity cDNA synthesis kit (Roche). Temporal and spatial gene expression patterns were examined by reverse transcript PCR (RT-PCR) using the NEB standard Taq polymerase system with the obtained cDNA of different tissues and development stages as a template. A 570 bp* Dicer1* fragment was amplified using forward: 5′ CAGAATAAAGATTTAGCGAATGG 3′ and reverse 5′ CTGCTTCTCCGGTGGTAG 3′ primers. Beta actin has been used as a house keeping gene and the primers forward: 5′ TGCCATGTATGTGGCCATCCA 3′ and reverse: 5′ AACACAGTGCTGTCTGGAGGT 3′ were used to amplify a 517 bp fragment. Gel electrophoresis was carried out on a 1% agarose gel.

### 2.7. Histology and Ultrastructure of Zebrafish Retina

Zebrafish were sacrificed using Schedule 1 method. Eyes from wild type and heterozygous Dicer mutant zebra fish at age of 20 months were fixed in 2.5% glutaraldehyde in 0.1 M phosphate buffer (PBS). The eyes were washed in 0.1 M PBS (15 minutes ×3) then fixed in 1% osmium tetroxide in 0.1 M PBS. The eyes were washed with distilled water and dehydrated (15 minutes ×3) in the graded series of ethanol (50% to 100%) and acetone (100%). The eyes were then infiltrated with a mixture of acetone + spur resin (50 : 50) for one hour and then into 100% resin (for 8 hours ×3). The eyes were polymerized into spur resin ant 70°C for 8 hours. Ultrathin sections were stained with uranyl acetate and lead citrate and observed under transmission electron microscope JEOL 1400. Digital micrographs were taken by side mounted* Valita* and bottom mounted* Quamisa* camera.

## 3. Results

### 3.1. Expression of Zebrafish* Dicer1*

**Figure 1:**
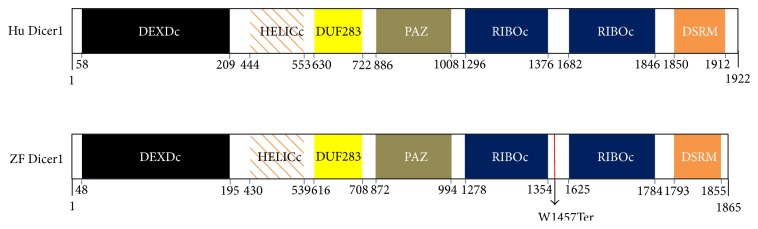
Schematic structure of human (Hu) and zebrafish (ZF) DICER1 proteins. Both human and zebrafish DICER1 have similar functional domains: N-terminal helicase domains (DEXDc and HELICc), Dicer dimerization domain (DUF283), PAZ domain, two ribonuclease III C terminal domains, and the double-stranded RNA binding motif (DSRM). The W1457Ter mutation was shown.

Zebrafish* Dicer1* encodes an open reading frame of 1865 amino acids and consists of at least 27 exons, spanning ~43 kb of genomic sequence on chromosome 17. Zebrafish DICER1 has similar functional domains to that of human DICER1 (Figure 1). Alignment of DICER1 protein sequences from zebrafish and other species exhibited that zebrafish DICER1 is highly homologous to other vertebrate species and less homologous to invertebrate and yeast. The functional domains of zebrafish DICER1 are also strongly conserved across vertebrate species. The helicase superfamily c-terminal domain (HELICc) and the second ribonuclease III C terminal domain (RIBOc) are conserved across vertebrate, invertebrate, and yeast species. The PAZ domain and the double-strand RNA binding motif (DSRM) are conserved across vertebrate and invertebrate species but not in yeast (data not shown).

**Figure 2:**
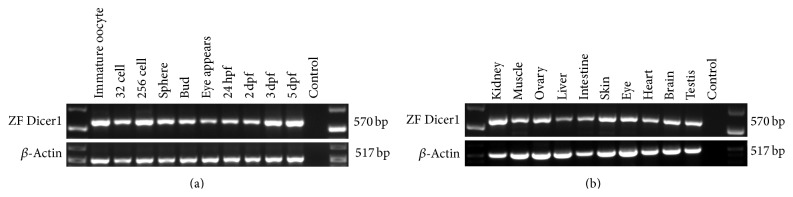
Expression of zebrafish* Dicer1*. (a) Temporal expression of zebrafish* Dicer1* transcripts detected by RT-PCR from total RNA extracted from oocytes and at different developmental stages. (b) Zebrafish* Dicer1* expression in different adult tissues detected by RT-PCR.

The temporal and spatial expression pattern of zebrafish* Dicer1* gene during embryogenesis, RT-PCR was carried out; zebrafish* Dicer1* mRNA was readily detected at the time of fertilization and persisted during gastrulation and through the tailbud and larval stages (Figure 2(a)). Zebrafish* Dicer1* expression in adult tissues was examined in total RNAs from zebrafish testis, brain, heart, eye, skin, intestine, liver, ovary, muscle, and kidney by RT-PCR. Zebrafish Dicer1 expression was readily detected in the eye and was detected in other tissues (Figure 2(b)).

### 3.2. Structure of Dicer Mutant Zebrafish Retina

**Figure 3:**
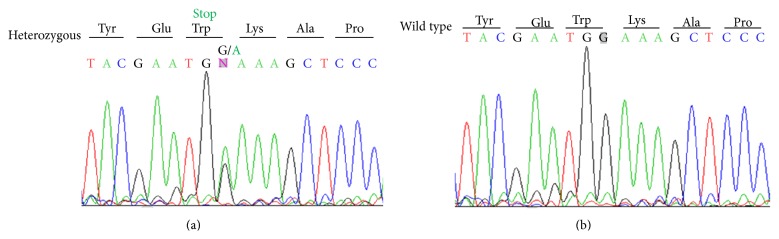
Sequence analysis of* Dicer1* genomic DNA in wild type zebrafish (b) and heterozygous zebrafish (a), with predicted changes to the sequence.

**Figure 4:**
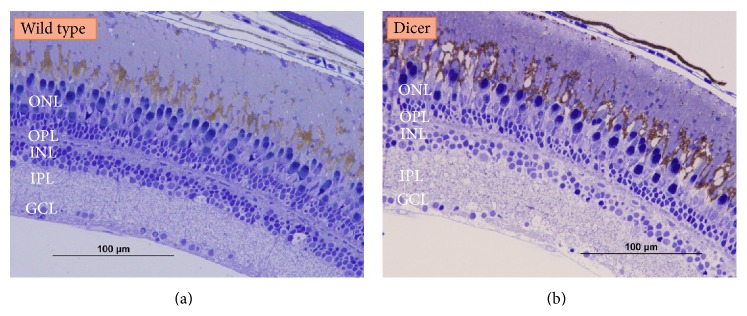
Light micrograph of histology of retina of normal and* Dicer1* mutant zebrafish. Light micrograph of part of retina showing retinal layers of wild type zebra fish (a); Light micrograph of part of retina showing retinal layers of* Dicer1* heterozygous mutant zebrafish (b). ONL: outer nuclear layer; OPL: outer plexiform layer; INL: inner nuclear layer; IPL: inner plexiform layer; GCL: ganglion cell layer.

All the homozygous zebrafish DICER1^W1457Ter/W1457Ter^ fish died on 14–18 dpf because of the general arrest of growth caused by the depletion of DICER1. The eyes of those homozygous mutant fish are significantly smaller than those of age-matched wild type fish at age of 7 dpf. Here we focused on characterising the aged heterozygous DICER1^WT/W1457Ter/+^ fish (Figure 3). Light microscopy observation showed that similar to wild type zebrafish, DICER1^WT/W1457Ter/+^ retina contained an outer nuclear layer, outer plexiform layer, inner nuclear layer, inner plexiform layer, and ganglion cell layer (Figure 4). No abnormality was observed in each layer.

**Figure 5:**
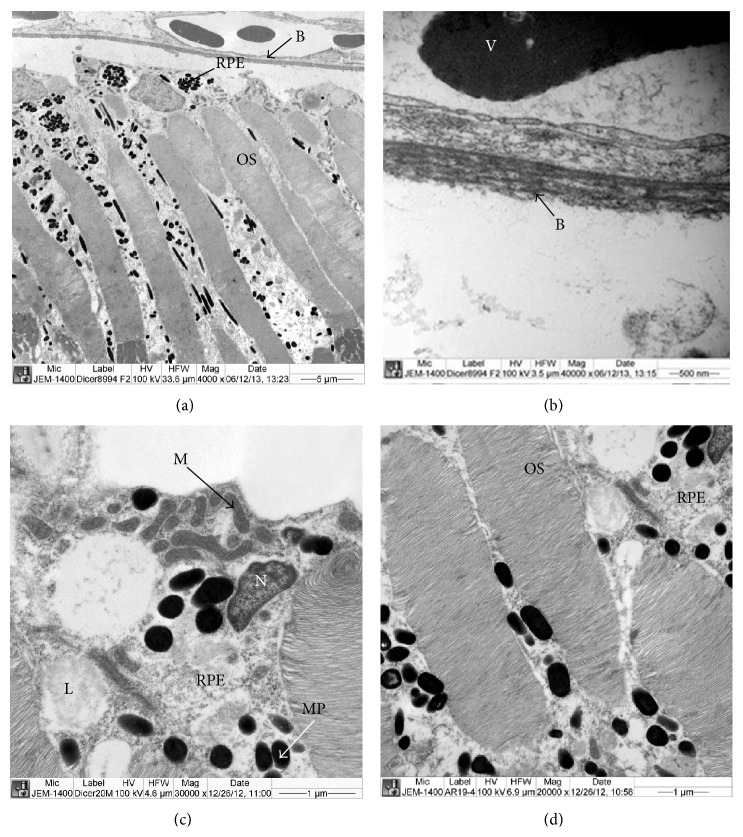
Ultrastructure of RPE cells and outer segment of photoreceptor of* Dicer1* mutant zebrafish. (a) Part of Bruch's membrane and RPE cells interdigitate with outer segments (OS) of photoreceptor. The RPE cells contained a large nucleus and melanin pigmented granules. Drusen were not present above the Bruch's membrane. (b) Part of the Bruch's membrane showing the normal fibrillar structure. Drusens were not present above the Bruch's membrane. (c) Part of the outer segment (OS) and RPE containing large nucleus, lysosome, mitochondria, and melanosomes (melanin pigmented granules). (d) Part of the outer segment showing regular disc lamellae. B: Bruch's membrane, GCL: ganglion cell layer, INL: inner nuclear layer, IPL: inner plexiform layer, L: lysosome, M: mitochondria, MP: melanosome (melanin pigmented granules), N: nucleus, ONL: outer nuclear layer, OS: outer segment, PL: photoreceptor layer, RPE: retinal pigmented epithelial cells.

Ultrastructural studies showed that the structure of the retinal pigmented epithelial cells (RPE) of heterozygous* Dicer1* zebrafish was similar to the structure of the RPE cells of age-control zebrafish described by Tarboush et al. [17]. Upper parts of the RPE cells were interdigitated with the outer segment of the photoreceptor and the lower parts of the RPE cells were extended between the outer segments of the photoreceptors (Figure 5(a)). The spindle shape or rounded shape melanin pigmented granules (melanosome) were dispersed in the apical and extended-part of the RPE cells (Figure 5(a)). Above the RPE, Bruch's membrane and blood vessels were observed (Figures 5(a) and 5(b)). The Bruch's membrane had a normal fibrillar structure and did not contain any drusen, a key feature of AMD (Figures 5(a) and 5(b)). The RPE cells contained a large nucleus, mitochondria, melanosomes, and lysosomes (Figure 5(c)). In most of the cones the lamellae were regularly stacked (Figure 5(d)).

**Figure 6:**
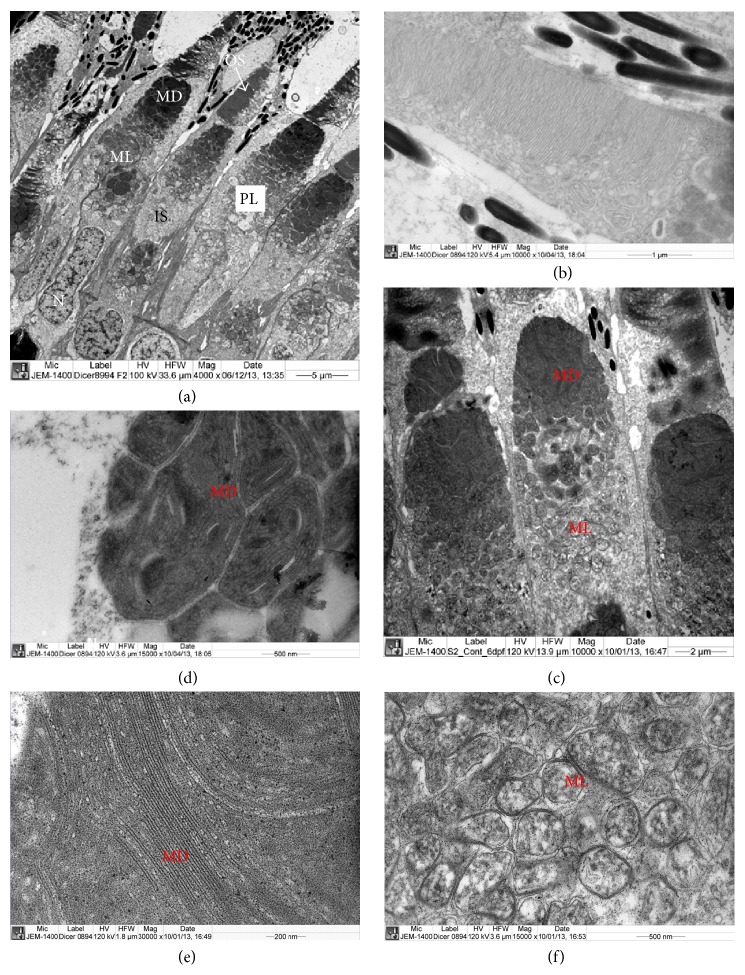
Ultrastructure of the photoreceptor layer of retina of* Dicer1* mutant zebrafish. (a) Part of the photoreceptor layer showing inner and outer segments of cones and rods. The inner segment contains electron dense mega-mitochondria (MD), electron lucent mega-mitochondria (ML), and nucleus. (b) Part of the outer segment containing disc lamellae. (c) Magnified image inner segment containing electron lucent and electron dense mega-mitochondria and large nucleus. (d) Magnified image of MD containing electron dense cisternae. (e) Magnified image of cisternae of MD containing electron lucent spaces between cisternae. (f) Magnified image of ML containing electron lucent cisternae. IS: inner segment, MD: electron dens mega mitochondria, ML: electron lucent mega mitochondria, N: nucleus, OS: outer segment, PL: photoreceptor layer.

**Figure 7:**
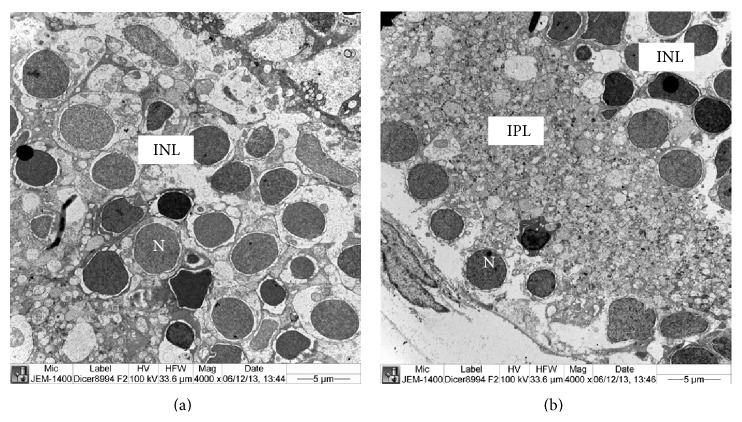
Ultrastructure of inner nuclear layer and ganglion cell layer of retina of* Dicer1* mutant zebrafish. (a) Part of the inner nuclear layer showing large nuclei. (b) Part of inner plexiform layer and ganglion cell layer containing large nuclei. GCL: ganglion cell layer, INL: inner nuclear layer, IPL: inner plexiform layer, N: nucleus.

The ultrastructure of rods and cones of retina of heterozygous* Dicer1* zebra fish was similar to the structure of rods and cones of retina of normal zebrafish described by Tarboush et al. [17]. The rods consisted of outer segment and inner segment (Figure 6(a)). The outer segment contained parallel disc lamellae (Figure 6(b)) while the inner segment contained large nucleus, electron dense and electron lucent mega mitochondria (Figures 6(a) and 6(c)). The electron dense mega mitochondria consisted of electron dense cisternae which enclosed very narrow electron lucent spaces (Figures 6(d) and 6(e)). The electron lucent mega mitochondria contained cisternae which are surrounded by large electron lucent spaces (Figure 6(f)). The inner nuclear layer, inner plexiform, layer and ganglion cell layers were also similar to those of the normal zebra fish layers (Figures 7(a) and 7(b)). Large nuclei were present in the ganglion cells (Figure 7(b)).

## 4. Discussion

DICER proteins have been identified in most eukaryotes, for example, animals, plants, and fungi. All DICER proteins reported to date have two RNase III domains. Dicers of higher species generally contain multiple functional domains, but lower eukaryotes frequently have few functional domains, for example, Trypanosoma Dicer has two RNase III domains only [18]. Zebrafish Dicer1 has a complex domain organization, similar to that of other vertebrate species (Figure 1 and data not shown). The zebrafish* Dicer1* gene encodes a protein of 1865 amino acids, which is highly homologous to the Dicer1 proteins identified in other vertebrate species (76%–81% amino acid identity) but shows lower homology to Dicer1 transcripts identified in invertebrate species (31%–49% identity). Analyses of zebrafish* Dicer1* during zebrafish development showed that* Dicer1* is highly expressed in oocytes, early cleavage stage embryos, and at late stage of development. The results are consistent with the recent findings of porcine* Dicer1* that was expressed during embryogenesis [19]. In the adult zebrafish,* Dicer1* expression was observed in all the tissues examined. The expression patterns of* Dicer1* in development and tissues suggest that* Dicer1* has a widespread role in tissue development and maintenance.

DICER1 is required for the production of small RNA molecules (siRNAs and miRNAs) that regulate gene expression [18]. Mouse embryonic stem cells with Dicer1 depletion were defective in differentiation* in vitro* and* in vivo* [20],* Dicer1* complete knock-out mice die before axis formation [21], suggesting that miRNAs play a critical role in mammalian early development. Conditional knock-out of* Dicer1* in mouse retina resulted in abnormal retinal cell differentiation [9, 10, 22]. Inhibition of three miRNAs, let-7, miR-125, and miR-9, caused similar defects in retinal development shown in* Dicer1* conditional knock-out mice, further confirming that miRNAs are essential for early retinal development [23]. Zebrafish* Dicer1* mutant fish, DICER1^W1457ter/W1457Ter^, are not embryonic lethal and go through early developmental stages, presumably due to the function of maternal Dicer1. This is supported by the observation that morpholino knock-down of* Dicer1* caused an earlier developmental arrest [5]. Depletion of both maternal and zygotic zebrafish DICER1 resulted in severe early embryonic morphogenesis, affecting gastrulation, somitogenesis, heart, and neural development [24]. Early eye development was highly delayed in DICER1^W1457ter/W1457Ter^ mutant fish, which presented with very small eyes, compared to those of wild type fish. The heterozygous mutant fish, DICER1^WT/W1457Ter^, did not show any abnormal eye development or any retinal degeneration (Figure 4).

Kaneko et al. showed DICER1 had the ability to degrade toxic RNA molecules [12], which might be involved in the pathogenesis of AMD. AMD is the most common cause of blind registration in the aged population, characterized by a late-onset degeneration of macula. AMD is likely to be a complex disease with the involvement of environmental and genetic factors. Late AMD occurs in two types: dry AMD with geographic atrophy and wet AMD with choroidal neovasculation [25]. In the RPE cells from dry AMD patients, DICER1 protein level was less than that of RPE cells from controls, but the abundance of* Alu* RNA was significantly increased compared to control RPE cells. DICER1 can degrade* Alu* RNA* in vitro* and* in vivo*, suggesting that reduced DICER1 level leads to the accumulation of Alu RNA and subsequent degeneration of RPE cells in dry AMD patients. Depletion of Dicer1 in mouse RPE cells caused RPE degeneration, which is similar to that in dry AMD patients. Knockdown of* Dicer1* in human RPE cells induced accumulation of* Alu* RNA which caused cytotoxicity [12]. Kaneko et al. did not examine whether there is any RPE degeneration in Dicer1 heterozygous knock-out mice. The* Dicer1* heterozygous mutant fish (DICER1^WT/W1457Ter^) are supposed to have 50% DICER1 protein in RPE cells. Since the function of DICER1 is conserved in evolution, decreased DICER1 protein level might also cause RPE degeneration in aged zebrafish. However, ultrastructural examination of the heterozygous mutant zebrafish RPE cells did not show any degeneration. Since AMD is a complex disease, both genetic and environmental factors contribute to the progression of the disorder [25]. The complement factor H (CFH) is a major AMD susceptibility gene for AMD, the Y402H substitution is associated with AMD risk [26–28]. Alignment of both human and zebrafish CFH proteins revealed that the human CFH Y402H variant is not conserved in zebrafish CFH (data not shown). So it is possible that the genetic difference results into no RPE degeneration in the heterozygous* Dicer1* mutant fish. It is also possible that zebrafish RPE cells have different microenvironment from mammalian RPE cells. Answers for the above questions require further investigation.
